# Editorial: Metallic alloys in medical applications

**DOI:** 10.3389/fbioe.2022.1041295

**Published:** 2022-10-06

**Authors:** Liqiang Wang, Lechun Xie, Daixiu Wei

**Affiliations:** ^1^ State Key Laboratory of Metal Matrix Composites, School of Materials Science and Engineering, Shanghai Jiao Tong University, Shanghai, China; ^2^ Hubei Key Laboratory of Advanced Technology for Automotive Components, Wuhan University of Technology, Wuhan, China; ^3^ Hubei Collaborative Innovation Center for Automotive Components Technology, Wuhan University of Technology, Wuhan, China; ^4^ Institute for Materials Research, Tohoku University, Sendai, Japan

**Keywords:** Biomaterials, biomedical, Bioengineering, Biotechnology, metal alloy

Biomaterials are natural or artificial materials used to manufacture implants to replace lost or diseased biological structures in order to restore form and function. Biomaterials that require long-term service, such as orthopedic implants, need good physical and chemical stability, such as stainless steel and titanium alloys. While biomaterials that need to disappear after a certain period of time such as vascular stents need biodegradability, such as magnesium alloys. In recent years, biomedical high entropy alloys (HEAs) as novel alloy systems are considered to have a greater potential in the biomedical field. In addition, there is an urgent need to use simulation and computation in the design and fabrication process of metallic implants for individual patient requirements.

In terms of traditional biomedical metals such as 316L stainless steel (316L SS) and Mg alloy, Li et al. prepared several 316L SS specimens with different angles (0°, 15°, 30°, 45°, 60°, 75°, and 90°) relative to the substrate by selective laser melting (SLM) and investigated the effects of different angles on the microstructure evolution, tensile properties, and corrosion resistance of 316L SS. The specimens with 90° relative to the substrate show higher toughness and corrosion resistance due to a higher volume fraction of low-angle grain boundaries and finer grains. Zhang et al. reviewed the recent developments in Mg alloys in optimizing composition and microstructure, enhancing mechanical properties, controlling degradation rates, and elucidating corrosion mechanisms. They pointed out that surface modification techniques should be utilized to improve the corrosion resistance of Mg alloys on the basis of ensuring biocompatibility and mechanical properties. Yang et al.prepared a mixture of metallic Fe and Mg in a layered composite using a one-step dip-coating method and evaluated the morphology, composition, crystal structure, and corrosion behavior of Mg/Fe sheets. Long-term open-circuit potential measurements showed that the Mg/Fe sheet specimens exhibited a “self-healing” effect in Dulbecco’s modified Eagle medium, which provides a new method to control the corrosion rate of Mg/Fe. Åhman et al. investigated the effect of powder bed laser melting and subsequent hot isostatic pressing (HIP) on the microstructure and weave of the specimens in different orientations and explored the corrosion behavior of Mg-Y-Nd-Zr alloys compared to the extruded specimens. The results show that more and coarser secondary phases lead to higher local corrosion rates in the powder bed laser melt specimens and HIPed specimens. Yang et al. prepared Mg-Ti composites with a bicontinuous interpenetrating phase structure by infiltrating Mg melt into 3D printed Ti scaffolds and explored its degradation behavior and effects on mouse embryonic osteoblast precursor cells. They found that Mg-Ti composite preferentially started to degrade near the interface and significantly enhanced osteogenic activity due to the positive effect of a moderate amount of Mg^2+^.

In the aspect of novel alloy systems, for example, Liu et al. summarized the superior mechanical properties and biocompatibility of biomedical HEAs. Moreover, biomedical HEAs show both high strength and low elastic modulus, which can meet the strength requirements and avoid stress shielding, and some of them exhibit good superelasticity. Regarding the biological properties, biomedical HEAs focus on the elements with good biocompatibility, such as Ti, Ta, Nb, Zr, and Hf. However, the biological behaviors of biomedical HEAs are currently limited to *in vitro* cell viability experiments, and relevant *in vivo* experiments still need to be carried out. Feng et al. summarized the composition of biomedical HEAs in recent years, introduced their biocompatibility and the mechanical properties matching with human bone, and gave suggestions for future directions. They concluded that there is a demand for advancing theoretical and simulation studies on the compositional design of biomedical HEAs, quantifying the effects of composition, process, and post-treatment on the performance of biomedical HEAs, as well as focusing on the depletion of biomedical HEAs under actual use conditions.

To meet individual service requirements, multi-level simulation and computation are utilized in the design of biomedical metallic implants. Hou et al. systematically investigated the phase stability, elastic modulus, and electronic structure of the body-centered cubic structure of β-TiX (X = Nb, Ta) alloy with the aid of first-principles calculations. The results show that the bonding strength between Ti and X atoms increases with the increase of the alloying element X content, which leads to an increase in phase stability and elastic modulus. Lv et al*.* designed porous scaffolds based on triply periodic minimal surface structures with the same porosity and different pore strategies by changing the minimal surface equation. They systematically investigated the effects of the pore strategy on the microstructure, mechanical properties, and permeability of the porous scaffolds, and the results showed that the continuous gradient distribution of pore size altered the stress distribution inside the scaffolds. Liang et al. proposed a reasonable cervical intervertebral range of motion after anterior cervical discectomy and fusion (ACDF) to offset the increased intervertebral facet joint forces and intradiscal pressures in the non-fusion segment after anterior cervical discectomy fusion. This study provides a new method to estimate reasonable range of motion after ACDF surgery from a biomechanical perspective, and further *in vitro* and clinical studies are needed for validation.

The contributions of the above work were summarized as follows: the traditional biomedical metals, especially 316L SS and Mg alloys (Li et al.; Zhang et al.; Yang et al.; Åhman et al.; Yang et al.); novel alloy system, biomedical HEAs (Liu et al.; Feng et al.); and simulation and computational methods (Hou et al.; Lv et al.; Liang et al.). This Research Topic focused on the recent development of metallic biomaterials for medical applications, which can promote the development of metallic biomaterials.

